# Reelin Proteolysis Affects Signaling Related to Normal Synapse Function and Neurodegeneration

**DOI:** 10.3389/fncel.2016.00075

**Published:** 2016-03-29

**Authors:** April L. Lussier, Edwin J. Weeber, G. William Rebeck

**Affiliations:** ^1^Department of Molecular Pharmacology and Physiology, USF Health Byrd Alzheimer’s Disease Institute, University of South FloridaTampa, FL, USA; ^2^Department of Neuroscience, Georgetown UniversityWashington, DC, USA

**Keywords:** Reelin, Alzheimer’s disease, neurodegeneration, proteolysis, learning and memory

## Abstract

Reelin is a neurodevelopmental protein important in adult synaptic plasticity and learning and memory. Recent evidence points to the importance for Reelin proteolysis in normal signaling and in cognitive function. Support for the dysfunction of Reelin proteolysis in neurodegeneration and cognitive dysfunction comes from postmortem analysis of Alzheimer’s diseases (AD) tissues including cerebral spinal fluid (CSF), showing that levels of Reelin fragments are altered in AD compared to control. Potential key proteases involved in Reelin proteolysis have recently been defined, identifying processes that could be altered in neurodegeneration. Introduction of full-length Reelin and its proteolytic fragments into several mouse models of neurodegeneration and neuropsychiatric disorders quickly promote learning and memory. These findings support a role for Reelin in learning and memory and suggest further understanding of these processes are important to harness the potential of this pathway in treating cognitive symptoms in neuropsychiatric and neurodegenerative diseases.

Neurodegenerative diseases are characterized by the progressive loss of synapses and neurons, accounting for cognitive deterioration. One molecular pathway that is well characterized in playing a role in adult synaptic plasticity and learning and memory is the Reelin signaling pathway (Weeber et al., 2002; Chen et al., 2005; Qiu et al., 2006a,b; Qiu and Weeber, 2007; Rogers et al., 2011). Reelin is also involved in a number of neurodegenerative and neuropsychiatric disorders presenting with cognitive deficits, including schizophrenia (Guidotti et al., 2000; Chen et al., 2002; Fatemi, 2005; Torrey et al., 2005), bipolar disorder (Fatemi et al., 2000; Torrey et al., 2005), depression (Knable et al., 2004; Lussier et al., 2009, 2011, 2013a,b; Fenton et al., 2015), epilepsy (Fournier et al., 2010; Haas and Frotscher, 2010; Dutta et al., 2011) and autism (Fatemi et al., 2005). Furthermore, Reelin signal transduction pathways appear to be particularly vulnerable in Alzheimer’s disease (AD), potentially contributing to its pathogenesis (Hoe et al., 2006; Hoareau et al., 2008). Thus, a better understanding of Reelin signaling could be useful in developing therapies against synaptic and neuronal loss in a number of conditions.

## Reelin in Development

Reelin is an extracellular matrix protein important in brain development during embryogenesis (for detailed reviews, see Lambert de Rouvroit et al., 1999; Rice and Curran, 2001; Tissir and Goffinet, 2003). During development Reelin is expressed by Cajal–Retzius cells in the hippocampus and cortex and granule cells in the cerebellum (Ogawa et al., 1995; Del Río et al., 1997; Frotscher, 1998; Hirota et al., 2015). In the adult brain GABAergic interneurons in the cortex and hippocampus secrete Reelin (Alcantara et al., 1998; Pesold et al., 1998). Much of what we know about the Reelin signaling pathway in development comes from mouse models that have knock-down or overexpression of critical proteins in the pathway: Reelin, lipoprotein receptors, and Disabled-1 (Dab1; Howell et al., 1997b; Hiesberger et al., 1999; Trommsdorff et al., 1999; Beffert et al., 2002; Drakew et al., 2002; Weeber et al., 2002; Qiu et al., 2006a; Pujadas et al., 2010, 2014; Teixeira et al., 2011; Trotter et al., 2013; Lane-Donovan et al., 2015).

## Reelin Signaling Pathway

**Figure 1:**
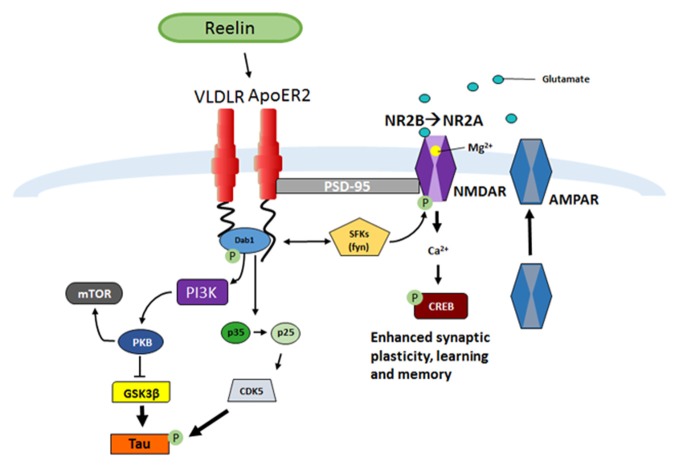
**Reelin signaling pathway in adult synaptic plasticity.** Reelin binds to the lipoprotein receptors apolipoprotein receptor 2 (ApoER2) and very-low-density lipoprotein receptor (VLDLR) which causes receptor clustering and the src family tyrosine kinases (SFK) tyrosine phosphorylation of the intracellular adaptor protein Disabled-1 (Dab1), which results in the phosphorylation of N-methyl-D-aspartate receptor (NMDAR; D’Arcangelo et al., 1997, 1999; Weeber et al., 2002; Niu et al., 2004; Beffert et al., 2005; Chen et al., 2005; Qiu et al., 2006b; Qiu and Weeber, 2007; Burrell et al., 2014; Divekar et al., 2014). A subsequent increase in calcium influx leads to depolarization of the post-synaptic membrane and α-amino-3-hydroxy-5-methyl-4-isoxazolepropionic acid receptor (AMPAR) insertion (Weeber et al., 2002; Qiu et al., 2006b; Qiu and Weeber, 2007). A consequence of the increase in Ca^2+^ influx and depolarization of the cell is increased CREB phosphorylation and protein synthesis, which ultimately results in increased synaptic plasticity and learning and memory (Niu et al., 2008; Rogers et al., 2011, 2013). Another result of Dab1 phosphorylation is activation of phosphatidylinositol-3-kinase (PI3K), protein kinase B (PKB/Akt), and modulation of Glycogen synthase kinase 3 beta (GSK3β), which inhibits Tau phosphorylation (Beffert et al., 2002). Phosphorylation of Dab1 also regulates the conversion of p35 to p25 and results in activation of CDK5, also responsible for Tau phosphorylation (Beffert et al., 2004).

Once Reelin is secreted by GABAergic interneurons into the extracellular space it binds to the lipoprotein receptors, very-low-density lipoprotein receptor (VLDLR) and Apolipoprotein receptor 2 (ApoER2; D’Arcangelo et al., 1999; Weeber et al., 2002; Herz and Chen, 2006; Figure 1). Ligand interactions lead to receptor dimerization and tyrosine phosphorylation of the downstream intracellular adaptor protein Dab1 (Howell et al., 1997a; D’Arcangelo et al., 1999; Hiesberger et al., 1999; Strasser et al., 2004; Herz and Chen, 2006; Trotter et al., 2013, 2014; Divekar et al., 2014). Dab1 phosphorylation activates Src family tyrosine kinases (SFK), such as Fyn, which phosphorylates N-methyl-D-aspartate (NMDA) receptors allowing increases in Ca^2+^ influx (Chen et al., 2005). Enhancement in Ca^2+^ influx allows for maturation of NMDA receptors from the NR2B to NR2A receptor subtype, increased membrane α-amino-3-hydroxy-5-methyl-4-isoxazolepropionic acid (AMPA) receptor insertion, and can contribute to the induction and enhancement of long-term potentiation (LTP; Weeber et al., 2002; Beffert et al., 2005; Chen et al., 2005; Herz and Chen, 2006; Qiu et al., 2006b; Qiu and Weeber, 2007). In addition, Dab1-induced phosphorylation also can activate Phosphatidylinositol-3-kinase (PI3K) and protein kinase B (PKB/Akt) which then causes Glycogen synthase kinase 3 beta (GSK3β) inhibition (Beffert et al., 2002), in turn suppressing tau hyperphosphorylation (Ohkubo et al., 2003).

As Reelin positive cells are found in highest numbers in the CA1 stratum lacunosum and hilus, they are in prime locations to influence learning and memory, and neurogenesis, respectively. Indeed, Reelin has been shown to enhance synaptic plasticity and learning and memory (Weeber et al., 2002; Herz and Chen, 2006; Rogers and Weeber, 2008), as well as alter migration of adult born neurons (Zhao et al., 2007; Pujadas et al., 2010; Teixeira et al., 2012). In the hippocampus, extracellular Reelin accumulates in the stratum lacunosum (Pesold et al., 1999; Lussier et al., 2009) which makes it in a prime location to influence synaptic activity in the CA1 (Weeber et al., 2002; Herz and Chen, 2006; Rogers and Weeber, 2008). Endogenous cleavage of Reelin in these regions may be used to regulate Reelin’s effects on these processes.

## Reelin Processing

**Figure 2:**
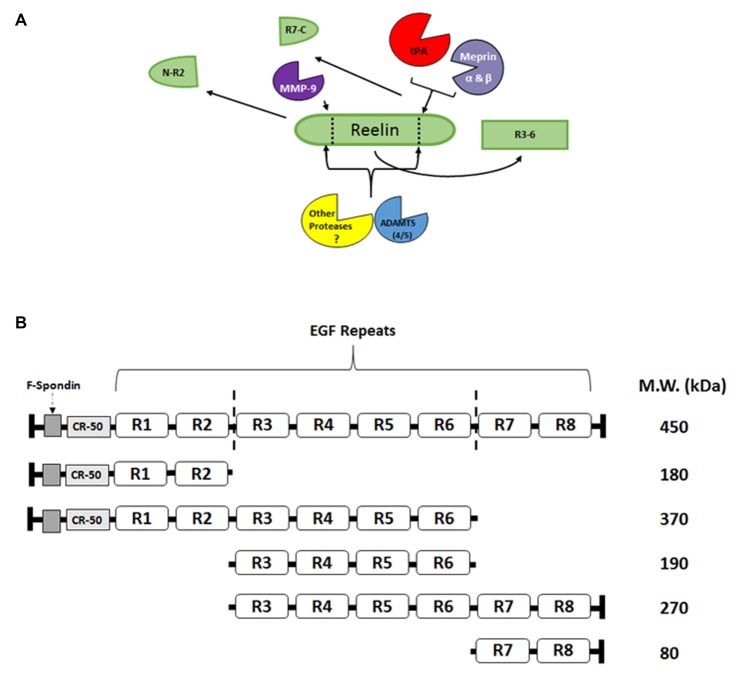
**Reelin proteolysis in the adult brain. (A)** Full length Reelin is released into the extracellular space by GABAergic interneurons in the adult brain. This full length Reelin is enzymatically cleaved between epidermal growth factor (EGF) repeats 2–3 (R2-R3) and 6–7 (R6-R7; indicated by dotted lines; **A,B**), by a number of different enzymes. For example, tissue plasminogen activator (tPA), Meprin α and β have been shown to cleave Reelin between R6 and R7 (Kohno et al., 2009; Krstic et al., 2012; Trotter et al., 2014; Sato et al., 2016), while matrix metalloproteinases (MMP)-9 cleaves Reelin between R2 and R3 (Krstic et al., 2012). The ADAMTS 4 and 5 have been shown to cleave Reelin at both sites (Hisanaga et al., 2012; Krstic et al., 2012). Other, yet to be identified proteases, are also potentially involved in Reelin processing. **(B)** Full length Reelin (450 kDa) is cleaved by a number of enzymes which result in the production of five fragments that range from 370–80 kDa. The R3-R6 fragment [included in the full length Reelin (450 kDa), 370 kDa, 190 kDa, and 270 kDa fragments] has been shown to bind to the lipoprotein receptors, ApoER2 and VLDLR (Jossin et al., 2004). The N-R2 fragment (180 kDa) has been shown to bind to alpha_3_beta_1_-integrins (Dulabon et al., 2000) and neuronal migration has been shown to be disrupted *in vivo* by the CR-50 antibody (Nakajima et al., 1997). The C-terminal region (R7-C; 80 kDa) has been shown to be involved the secretion of Reelin, as well as its proper folding (de Bergeyck et al., 1997; Jossin et al., 2004), and for downstream signaling efficacy (Nakano et al., 2007).

Reelin signaling may not be driven by the simple production and release of Reelin from interneurons, as with neuropeptides or small molecule transmitters, but it may be regulated by the directed proteolysis of sequestered, full length, extracellular Reelin. Reelin has been shown to have two main sites of cleavage, between EGF-like repeats 2–3 (R2–3) and repeats 6–7 (R6–7; Jossin et al., 2004; Figure 2). These cleavage sites result in five major fragments that can be found in the adult and developing brain (Jossin et al., 2007; Krstic et al., 2012; Trotter et al., 2014). The middle R3–6 fragment interacts with the VLDLR and ApoER2 and is considered the fragment that is involved in initiating the downstream signaling of the Reelin cascade (Jossin et al., 2004). Our laboratory (Trotter et al., 2014) and others (Nagy et al., 2006; Nogi et al., 2006; Nakano et al., 2007; Hisanaga et al., 2012; Krstic et al., 2012) have attempted to identify Reelin-cleaving enzymes, such as the serine protease tissue plasminogen activator (tPA), matrix metalloproteinases (MMP), and a disintegrin and metalloproteinase with thrombospondin motifs (ADAMTS), and the functional role of this proteolytic processing.

We have recently identified one mechanism for the normal processing of extracellular Reelin, through the effects of the serine protease tPA in the brain (Trotter et al., 2014). The activity-dependent proteolysis of Reelin between R6 and R7 in wild-type mice was not seen in tPA KO mice, supporting a role of this protease in NMDAR-independent LTP induced cleavage of Reelin (Trotter et al., 2014). In cell culture, Reelin cleavage between R6–7 by tPA was blocked by serpin E1 inhibitor (Krstic et al., 2012). Our cell-free conditions in which we incubated tPA with Reelin for 45 min also produced increased the N-R6 fragment (370 kDa), which was blocked with Plasminogen activator inhibitor (PAI-1; serpin E1) and diisoporpyl fluorophosphates (a serine protease inhibitor), but not blocked by Aprotinin or CR-50 (an antibody that binds in the N-terminal region of Reelin; D’Arcangelo et al., 1997; Trotter et al., 2014). Similarly, metalloproteases meprin α and β cleave Reelin between the R6 and R7 repeats (Sato et al., 2016). However, neither tPA knock-out mice (Trotter et al., 2014) nor meprin β knock-out mice (Sato et al., 2016) demonstrate differences in basal levels of full length Reelin or Reelin fragments, suggesting that combinations of proteases are involved in constitutive Reelin levels and proteolysis. Furthermore, Reelin proteolysis may be important in activity-dependent or pathological conditions.

Much of what is known about signaling abilities of specific Reelin domains comes from research on the canonical Reelin-lipoprotein-Dab1 pathway (Figure 1). In support of the importance of the middle R3–6 fragment in lipoprotein receptor binding, cleavage within the R3 repeat has recently been shown to decrease Dab1 phosphorylation (Kohno et al., 2009; Koie et al., 2014). However, the other fragments have also been suggested to be vital for normal Reelin signaling. For example, the N-R2 fragment has been shown to bind to alpha_3_beta_1_-integrins (Dulabon et al., 2000) and the CR-50 antibody can disrupt *in vivo* neuronal migration (Nakajima et al., 1997). The C-terminal region (R7-C) has been suggested to be involved in Reelin secretion, folding (de Bergeyck et al., 1997; Jossin et al., 2004), and signaling efficacy (Nakano et al., 2007), although no known receptors have been identified for R7-C binding. Recently, Kohno et al. (2015), have shown that the C-terminal region is critical in postnatal cerebral cortex development but not in embryonic stages. Further research is needed to fully elucidate the importance of these specific fragments in normal and pathological conditions.

## Reelin and Neuropsychiatric/Neurodegenerative Disorders

Support for the role of Reelin proteolysis in human disease has been found in both neuropsychiatric and neurodegenerative disorders. For example, the N-R2 fragment is increased in AD and frontotemporal dementia patients when compared to non-demented patients (Sáez-Valero et al., 2003; Botella-López et al., 2006). In patients with confirmed diagnosis for depression and bipolar disorder, the N-R2 fragment is found to be decreased in blood samples, while for schizophrenia patients the N-R6 fragment is increased (Fatemi et al., 2001). Reelin may also play a role in seizure control: epilepsy models have altered Reelin processing (Tinnes et al., 2011, 2013; Kaneko et al., 2016), which may be MMP-dependent. These differences in Reelin fragment levels point to an importance in Reelin levels and proteolytic dysfunction in disease states.

## Reelin and AD Pathoetiology

In AD, loss of synapses and neurons is accompanied neuropathologically by amyloid deposits composed of the Amyloid beta (Aβ) peptide, and neurofibrillary tangles composed of modified versions of the tau protein (Trojanowski and Lee, 2002; Schellenberg and Montine, 2012; Sheng et al., 2012). Exogenous Aβ application and endogenous Aβ aggregates block various forms of synaptic plasticity and inhibit memory formation and retrieval (Klyubin et al., 2005; Selkoe, 2008; Talantova et al., 2013). Hyperphosphorylated forms of tau are also associated with the disruption of synaptic plasticity, learning and memory (Trojanowski and Lee, 2002; Santacruz et al., 2005; Lasagna-Reeves et al., 2011, 2012; Shipton et al., 2011). Altered Reelin signaling has been linked to AD through analyses of human brain samples (Herring et al., 2012; Notter and Knuesel, 2013), and animal models connecting Reelin to the processes of amyloid accumulation (Chin et al., 2007; Kocherhans et al., 2010; Pujadas et al., 2014) and to tau phosphorylation (Ohkubo et al., 2003; Herz and Chen, 2006; Kocherhans et al., 2010; Cuchillo-Ibáñez et al., 2013). In addition, Reelin signaling has been associated with human AD synaptic dysfunction in a non-targeted transcriptomic approach (Karim et al., 2014), and the Reelin gene was associated with AD pathological findings in elderly controls in a non-targeted genomic approach (Kramer et al., 2011). Finally, two of the strongest genetic risk factors for AD, Apolipoprotein E (APOE) and clusterin (APOJ), encode proteins that bind to the Reelin receptors (Reddy et al., 2011; Tapia-González et al., 2011).

These lines of research have led to the investigation of possible mechanisms for how Reelin could specifically affect AD. Reelin may modify amyloid levels by directly interacting with amyloid precursor protein (APP; Hoe et al., 2009) or altering APP metabolism to decrease the generation Aβ (Rice et al., 2013; Pujadas et al., 2014). Reelin also causes GSK3β inhibition (Beffert et al., 2002), which suppresses tau hyperphosphorylation (Ohkubo et al., 2003). In mouse models of AD, overexpressing Reelin prevented AD pathological changes (Pujadas et al., 2014), and lowering levels of Reelin accelerated Aβ deposition and the synaptic dysfunction caused by the presence of amyloid (Kocherhans et al., 2010; Lane-Donovan et al., 2015). In addition to these effects on the neuropathologic accumulations in AD brain, several lines of evidence suggest that Reelin and Aβ have antagonistic effects on neuronal survival and signaling. These findings include reduction of Reelin and Reelin signaling in an AD mouse model (Mota et al., 2014), electrophysiological measures of Reelin and Aβ effects on hippocampal brain slices (Durakoglugil et al., 2009), and behavioral studies in AD mouse models with altered levels of Reelin (Pujadas et al., 2010; Lane-Donovan et al., 2015).

## Reelin as a Therapeutic Target

As mentioned above, different Reelin fragments are altered in neuropsychiatric and degenerative diseases. These alterations may be an indication of disruption in Reelin processing and may be useful in identifying biomarkers for disease states. Reelin has been shown to be sequestered by Aβ plaques in an age-dependent manner (Knuesel et al., 2009; Doehner and Knuesel, 2010; Kocherhans et al., 2010; Stranahan et al., 2011). Removal of Reelin from the synapse can alter many Reelin-dependent functions, causing abnormal cellular migration, dendritic morphology atrophy and deficits in synaptic plasticity (Herz and Chen, 2006; Rogers and Weeber, 2008; Bu, 2009). Given the progressive memory decline seen in AD patients, it is possible that the sequestering of Reelin by the amyloid plaques can alter its normal regulation via cleavage mechanisms and its normal enhancement of learning and memory. It is interesting to note that crossing a transgenic mouse that overexpresses Reelin with an AD mouse model protects from amyloid plaque formation and rescues learning and memory deficits when compared to the AD mice (Pujadas et al., 2014), while decreasing Reelin in AD models accelerates plaque formation and increases tau hyperphosphorylation (Kocherhans et al., 2010). Interestingly, a novel inducible Reelin knockout mouse line has revealed that adult knockdown of Reelin expression results in no discernable differences in normal learning and memory and actually enhances late LTP (Lane-Donovan et al., 2015). When these Reelin knockdown mice were crossed with Tg2576 AD mice they did not cause an increase Aβ pathology; however, these mice showed poorer learning in the hidden platform water maze and deficits in the 24 h probe test when compared to controls (Lane-Donovan et al., 2015). These results support the importance of Reelin signaling in normal cognitive function and shows that a loss of Reelin signaling in an AD mouse model increases cognitive dysfunction.

Introduction of exogenous Reelin into the brain can have surprisingly profound effects on synaptic plasticity and cognition. Hippocampal injection(s) of Reelin and its fragments has demonstrated significant improvements in models of Reelin deficiency (Rogers et al., 2013), Angelman syndrome (Hethorn et al., 2015), and schizophrenia (Ishii et al., 2015). Remarkably, exogenous Reelin also enhanced learning and memory as well as increased synaptic plasticity in wild-type mice (Rogers et al., 2011). Thus, therapeutic approaches to promoting Reelin signaling could be useful in protecting synapse function and survival in a range of disorders. This work would require a better understanding of which domains of Reelin are necessary for the regulated Reelin signaling, and assays for examining whether new Reelin-based therapies promote receptor clustering, intracellular signaling, synapse stabilization, and neuronal protection. Although more work is required to fully understand the function of each of these Reelin fragments, the current research points to a therapeutic potential for altering specific Reelin fragments in treating neuronal dysfunction and cognitive deficits in neurodegenerative and neuropsychiatric disorders.

## Author Contributions

ALL wrote the review and generated the figures. EJW and GWR edited the content and structure of the review. Each author contributed ideas of topics for inclusion.

## Conflict of Interest Statement

The authors declare that the research was conducted in the absence of any commercial or financial relationships that could be construed as a potential conflict of interest.
